# Using AlphaFold to predict the impact of single mutations on protein stability and function

**DOI:** 10.1371/journal.pone.0282689

**Published:** 2023-03-16

**Authors:** Marina A. Pak, Karina A. Markhieva, Mariia S. Novikova, Dmitry S. Petrov, Ilya S. Vorobyev, Ekaterina S. Maksimova, Fyodor A. Kondrashov, Dmitry N. Ivankov

**Affiliations:** 1 Center of Life Sciences, Skolkovo Institute of Science and Technology, Moscow, Russia; 2 Peoples’ Friendship University of Russia (RUDN University), Moscow, Russia; 3 Armand Hammer United World College of the American West, Montezuma, New Mexico, United Stated of America; 4 Specialized Educational and Scientific Center of UrFU (SUNC UrFU), Ekaterinburg, Russia; 5 Institute of Science and Technology Austria, Maria Gugging, Austria; 6 Evolutionary and Synthetic Biology Unit, Okinawa Institute of Science and Technology Graduate University, Onna, Okinawa, Japan; Berlin Institute of Technology, GERMANY

## Abstract

AlphaFold changed the field of structural biology by achieving three-dimensional (3D) structure prediction from protein sequence at experimental quality. The astounding success even led to claims that the protein folding problem is “solved”. However, protein folding problem is more than just structure prediction from sequence. Presently, it is unknown if the AlphaFold-triggered revolution could help to solve other problems related to protein folding. Here we assay the ability of AlphaFold to predict the impact of single mutations on protein stability (ΔΔG) and function. To study the question we extracted the pLDDT and <pLDDT> metrics from AlphaFold predictions before and after single mutation in a protein and correlated the predicted change with the experimentally known ΔΔG values. Additionally, we correlated the same AlphaFold pLDDT metrics with the impact of a single mutation on structure using a large scale dataset of single mutations in GFP with the experimentally assayed levels of fluorescence. We found a very weak or no correlation between AlphaFold output metrics and change of protein stability or fluorescence. Our results imply that AlphaFold may not be immediately applied to other problems or applications in protein folding.

## Introduction

AlphaFold is widely claimed to have revolutized protein 3D structure prediction from protein sequence, a 50-years long-standing challenge of protein physics and structural bioinformatics [1]. The fourteenth round of CASP, a blind competition on protein 3D structure prediction [2], demonstrated that AlphaFold, a newcomer to the field, significantly outperforms all other methods. Crucially, AlphaFold models showed an accuracy of their predicted structures that was comparable to structures solved by experimental methods, like X-ray crystallography, NMR, and Cryo-EM [3].

‘It will change everything’, said Andrei Lupas in an interview to Nature [3]. One of the primary changes may be that AlphaFold may also solve other problems related to protein folding. These problems include the prediction of various protein interactions, such as protein-protein, protein-ligand and protein-DNA/RNA, and the prediction of the impact of mutations on protein stability. AlphaFold proved to be useful for experimental determination of protein structures with molecular replacement phasing [4, 5] and already facilitated elucidation of SARS-Cov2 protein structures [6, 7]. Next, AlphaFold in collaboration with EMBL-EBI constructed the structure models for the whole protein sequence space [8]. The database of freely available structures of all proteins, is attributed to “revolutionize the life sciences” [3]. A pool of high-quality predicted structures is a plus for 3D-based prediction of mutation influence on protein stability since 3D-based prediction is more accurate than 1D-based one [9–11]. Furthermore, AlphaFold is expected to bring new insights into our understanding of the structural organization of proteins, boost the development of new drugs and vaccines [12]. Researchers in the field are already actively testing AlphaFold performance in various bioinformatics tasks, for instance, in peptide-protein docking [13, 14].

Guided by the expected immediate impact of AlphaFold for the solution of a wide range of problems in structural bioinformatics, we explored the capacity of AlphaFold predictions to serve as a proxy for the impact of mutations on protein stability change (ΔΔG). Although AlphaFold provides a disclaimer that it “has not been validated for predicting the effect of mutations” (https://alphafold.ebi.ac.uk/faq), the expectations of AlphaFold are so high that we judged it prudent to check how well AlphaFold predictions could work for estimation of ΔΔG values. Therefore, given that pLDDT score reflects confidence of the location of the residue in the structure, it may be expected that this measure correlates with ΔΔG or protein function. We found that the difference between pLDDT scores, the only local AlphaFold prediction metric reported in the output PDB file, had a very weak correlation with experimentally determined ΔΔG values (Pearson correlation coefficient, PCC = -0.17). The difference in the global AlphaFold metric—the pLDDT averaged for all residues—shows no correlation, both isolated and in combination with the mutated residue’s pLDDT score. Similarly, the same AlphaFold metrics had a very weak correlation with the impact of single mutations on protein function, fluorescence, of GFP. Recent results [15] show that the use of AlphaFold models instead of template structures does not improve ΔΔG prediction. Taken together, so far we failed to find a use for AlphaFold to predict the impact of a mutation on protein stability. The availability of AlphaFold models allows applying more accurate 3D protein structure-based ΔΔG predictors rather than sequence-based ΔΔG predictors; the bottleneck still seems to be the accuracy of current 3D protein structure-based ΔΔG predictors.

## Materials and methods

### Dataset of experimental mutations

The data on experimentally measured effects of mutations on protein stability were taken from ThermoMutDB [16] (version 1.3). From 13,337 mutations in the database we extracted single-point mutations with data on ΔΔG measured in the experimental conditions of pH between 3 and 9, and temperature between 293 to 300 Kelvins. We also put the restriction on protein length for it to be less than 250 amino acids. Since stabilizing mutations have to have negative ΔΔG while in ThermoMutDB they are positive, all ΔΔG values from ThermoMutDB were multiplied by −1.

The filtered dataset resulted in 1779 mutations in 80 proteins. We have done the analysis for randomly chosen 1154 mutations in 73 proteins. The final dataset and computed metrics are given in S1 Table.

### Dataset of GFP mutants fluorescence

We took data on fluorescence levels of GFP mutants from [17]. From the original dataset we randomly extracted 796 single mutants for our analysis. The list of the chosen mutations is given in S2 Table.

### Protein structure modeling with AlphaFold

The wild type protein structures were retrieved from the AlphaFold Protein Structure Database (AlphaFold DB) [8] by their UniProt accession code. The structures of original proteins that were absent in the AlphaFold DB as well as structures of mutant proteins were modeled by the standalone version of AlphaFold [1] using the fasta file with UniProt sequence of a protein as the only input in the ‘–fasta_paths’ flag.

### Prediction metrics

The per-residue local distance difference test (pLDDT) confidence scores for the protein structure models downloaded from the AlphaFold DB were retrieved from the B-factor field of the coordinate section of the pdb file. The pLDDT confidence scores for the protein structure models that we predicted by standalone AlphaFold were extracted from the pickle file, from “plddt” array. By default, AlphaFold produces five models. The differences in pLDDT and <pLDDT> were statistically significant within the group of five produced models (both for wildtype and mutant); we used in our analysis only the best one, i.e., having the highest value of <pLDDT>.

### Sequence identity of proteins within the dataset of mutations

To identify the sequence identities between the proteins in the dataset of mutations we performed protein BLAST [18] search of protein sequences against themselves.

We divided the dataset into training and test sets for linear regression model based on the arbitrary sequence identity threshold of 50%. Mutations in proteins above the threshold comprised the training set, and the rest of mutations were used as the test set. The training and test sets resulted in 423 mutations in 50 proteins and 731 mutations in 23 proteins, respectively.

### Linear regression analysis

Multiple linear regression fit with two parameters was performed using the linear_model module of Sklearn library with default parameters.

### Properties of mutated amino acid residues

Mutated amino acids were annotated by relative solvent accessibility, effect of mutation on stability, hydrophobicity, polarity, and side chain size.

Information on solvent accessibility was taken from Stride [19]. The relative solvent accessibility (RSA) of an amino acid residue was calculated according to the equation:

$$\begin{array}{c} RSA=\frac{ASA}{maxASA} \end{array}$$ (1)

where *ASA* is the solvent accessible surface area and *maxASA* is the maximum possible solvent accessible surface area of an amino acid [20]. Following [21] we used the solvent accessibility threshold of 25% to classify residues as exposed or buried.

The rest of the properties were assigned according to http://www.imgt.org/IMGTeducation/Aide-memoire/_UK/aminoacids/IMGTclasses.html. The side chain sizes were annotated as very small (1), small (2), medium (3), large (4), very large (5). We defined ‘no’, ‘small’, and ‘large’ change in size chain volume equal to difference of 0, 1 or 2, and 3 or 4 in absolute values, respectively.

All correlations were adjusted for multiple hypotheses testing by Benjamini-Hochberg correction [22].

## Results

### Data set of mutations

We used experimental data on protein stability changes upon single-point variations from ThermoMutDB Database [16]. After the filtering procedure (see Materials and methods) we performed analysis for 1154 mutations in 73 proteins. For the multiple linear regression analysis, the dataset was split into two sets, a training and a test set. The split was based on BLAST [18] results, such that the mutations were assigned to the test set if corresponding proteins had <50% sequence identity to any other protein in the entire dataset (see Materials and methods). All of the other mutations were assigned to the training set.

### AlphaFold prediction metrics

Along with coordinates of all heavy atoms for a protein, AlphaFold model contains “its confidence in form of a predicted lDDT-C*α* score (pLDDT) per residue” [1]. LDDT ranges from 0 to 100 and is a superposition-free metric indicating to what extent the protein model reproduces the reference structure [23]. The pLDDT scores averaged across all residues designate the overall confidence for the whole protein chain (<pLDDT>). The distributions of AlphaFold prediction metrics for wildtype and mutant structures statistically significantly differ from each other, both for pLDDT (p-value = 7 ⋅ 10^-10^) and <pLDDT> (p-value = 3 ⋅ 10^-3^). For each mutation in the dataset, we calculated the difference in pLDDT between the wild type and mutated structures in the mutated position as well as the difference in <pLDDT> between wild type and mutant protein structure models. By checking ΔpLDDT and Δ<pLDDT> values as potential proxies for the change of protein stability we explored the hypothesis that the change of protein stability due to mutation is somehow reflected in the difference of AlphaFold confidence between wild type and mutant structures.

### Correlation between ΔΔG and ΔpLDDT values

**Fig 1:**
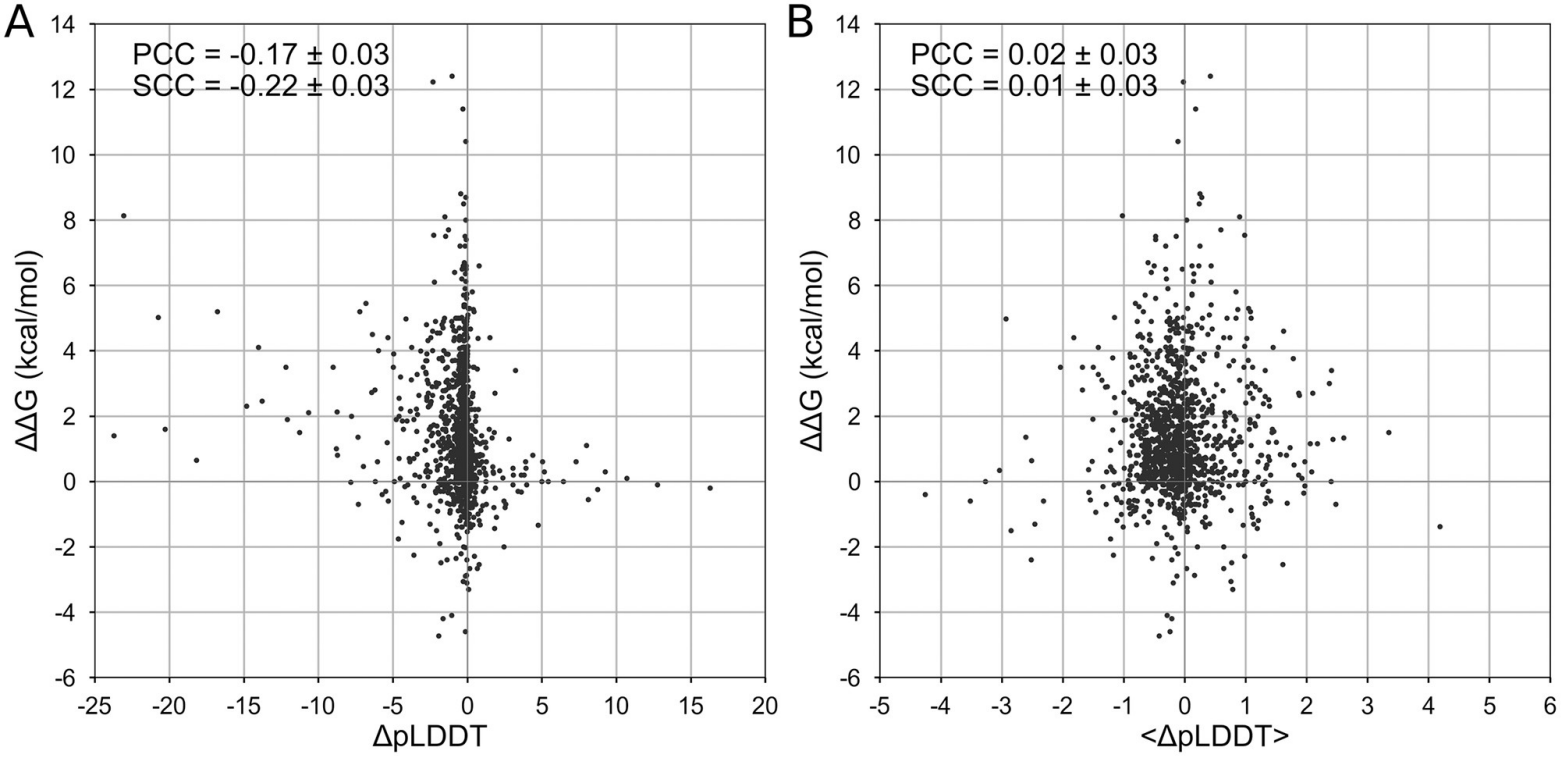
Correlation between ΔΔG and ΔpLDDT. Correlation between the effect of mutation on protein stability, ΔΔG, and change of confidence score of structure prediction, ΔpLDDT. A: The correlation for the mutated amino acid. B: The correlation for the whole structure.

First, we studied the relationship between the effect of mutation on protein structure stability and the difference in the accuracy of protein structure prediction by AlphaFold for the wild-type and mutant proteins. We did not observe a pronounced correlation between the mutation effect and the difference in confidence metrics (Fig 1). The correlation coefficient is -0.17 ± 0.03 (p-value = 10^-8^) for ΔpLDDT and 0.02 ± 0.03 (p-value = 0.44) for the Δ<pLDDT>.

Since the confidence metrics for a given amino acid and whole protein are weakly correlated (PCC = 0.21 ± 0.03, p-value = 10^-12^) we then explored how their combination correlates with the effect of mutation. Multiple linear regression model resulted in the dependence ΔΔG = -0.99–0.13 ⋅ ΔpLDDT + 0.03 ⋅ Δ<pLDDT>. We did not obtain any pronounced correlation either for training (0.12 ± 0.05, p-value = 0.01) or test sets (0.20 ± 0.04, p-value = 3 ⋅ 10^-8^).

### Relationship of ΔpLDDT and amino acid properties

We explored the outliers with high absolute values of ΔpLDDT in Fig 1A. Expectedly, the destabilizing effect of mutations was associated with decreasing pLDDTs: 87% of destabilizing mutations had negative ΔpLDDT (p-value = 10^−22^). However, there was no correlation between ΔpLDDT and ΔΔG for that 87% of mutations with negative ΔpLDDT.

We explored the correlation between AlphaFold prediction metrics and ΔΔG for different categories of mutations (see Methods, Properties of mutated amino acid residues). The correlation remained poor (|PCC| being less than 0.19 and 0.07 for ΔpLDDT and Δ<pLDDT>, respectively, see S3 Table) for mutations stratified by their effect on polarity, hydrophobicity, charge upon mutation and relative solvent accessibility of mutated residue (S3 Table).

The difference in pLDDT score distributions was significant for positions with the different secondary structures of mutated residue (Kruskal-Wallis p-value = 4 ⋅ 10^−10^) and for mutations changing the side chain size (Kruskal-Wallis p-value = 0.04). However, the correlation between ΔpLDDT or Δ<pLDDT> and ΔΔG for different types of mutations within these categories was not strong (|PCC| < 0.38 except for 29 mutations having a large increase in size showing correlation of -0.67 for ΔpLDDT, see S3 Table).

### Correlation between GFP fluorescence and ΔpLDDT values

**Fig 2:**
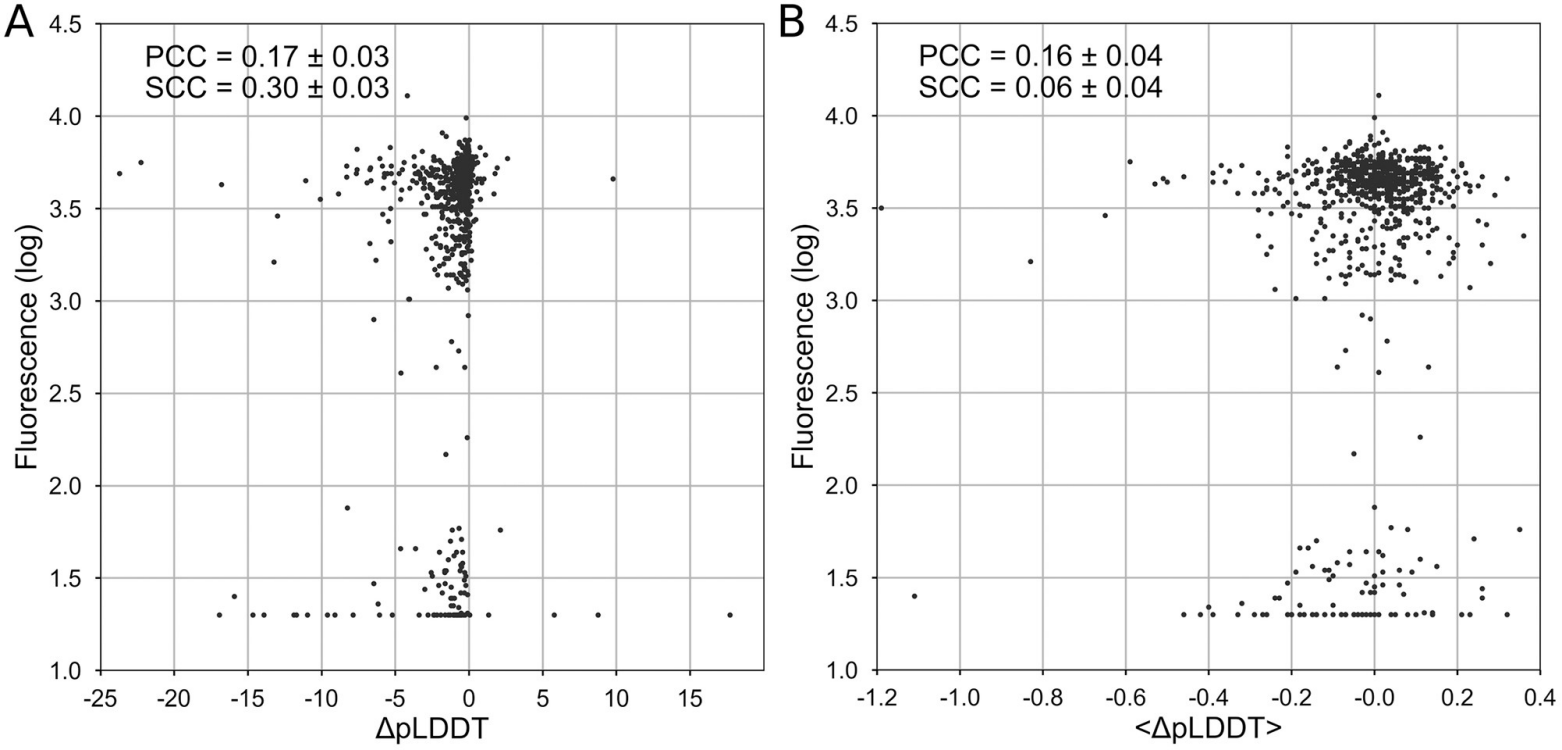
Correlation between the GFP fluorescence and ΔpLDDT. Correlation between the GFP fluorescence and change of confidence score of structure prediction, ΔpLDDT. A: The correlation for the mutated amino acid. B: The correlation for the whole structure.

Protein stability is intimately coupled with protein functionality. Thus, a reasonable hypothesis holds that the loss of protein functionality due to mutations in most cases results from reduced stability [24]. Therefore, along with testing correlation of AlphaFold metrics with ΔΔG, it is reasonable to test the correlation of AlphaFold metrics with protein function. Furthermore, the change of pLDDT scores may contribute directly to protein functionality without contributing to protein stability. We checked the correlation between ΔpLDDT values and the fluorescent level of 796 randomly chosen single GFP mutants from [17]. The correlation coefficient is 0.17 ± 0.03 (p-value = 3 ⋅ 10^-6^) for ΔpLDDT and 0.16 ± 0.04 (p-value = 10^-5^) for the Δ<pLDDT> (Fig 2).

## Discussion

Extraordinary success of AlphaFold in predicting protein 3D structure from protein sequence may lead to temptation to apply this tool to other questions in structural bioinformatics. Here we checked the potential of AlphaFold metrics to serve as a predictor for the impact of mutation on protein stability and function. We found a weak correlation of -0.17 ± 0.03 between ΔpLDDT and ΔΔG associated with specific mutations. Although the correlation was statistically significant (p-value < 10^-8^), it is so weak that it cannot be used for accurate ΔΔG predictions (Fig 1) and it is unclear how such predictions can be used in practical applications. Clearly, ΔpLDDT would show a better correlation with ΔΔG if it was measured across bins of averaged ΔΔG. Alternatively, ΔpLDDT could be a separate term in a multiple linear regression model. The averaged metric Δ<pLDDT> shows correlation with ΔΔG, which is statistically indistinguishable from zero. However, a linear combination of the two metrics, ΔpLDDT and Δ<pLDDT>, does not greatly improve the correlation. As for the loss-of-function prediction, the correlation with the impact of mutation on GFP fluorescence showed similar results: PCC was 0.17 ± 0.03 and 0.16 ± 0.04 for ΔpLDDT and Δ<pLDDT>, respectively (Fig 2).

Taken together, our data indicate that AlphaFold predictions cannot be used directly to reliably estimate the impact of mutation on protein stability or function. But why should we have expected such a correlation in the first place? Indeed, AlphaFold was not designed to predict the change of protein stability or function due to mutation. In the words of the authors “AlphaFold is not expected to produce an unfolded protein structure given a sequence containing a destabilising point mutation” (https://alphafold.ebi.ac.uk/faq). However, the only reason for a protein to fold into the distinct native structure is the stability of this structure, so the protein 3D structure and its stability are closely connected. Logically, an algorithm predicting protein 3D structure from sequence should search for the most stable 3D state under the native (or standard) conditions. If a compact structure becomes unstable (for example, due to mutation) then we might expect that the algorithm shifts its predictions toward an unfolded state. Evidence in favor of this point of view is the successful prediction of natively disordered protein regions by AlphaFold and the correlation between the decrease of pLDDT and propensity to be in a disordered region [25]. Thus, it is not unreasonable to expect a decrease in the confidence score of the mutated residue or the whole native structure.

Indeed, it was reported many times that 3D-based predictors perform better than 1D-based [9–11], so the availability of a pool of high-quality 3D predicted structures could be a plus.

Our results show that AlphaFold repurposing for ΔΔG prediction did not work for the proteins we studied. AlphaFold 3D models can be used to predict the impact of a mutation on protein stability or function by 3D-structure-based ΔΔG predictors. However, the performance of the resulting predictions is going to be far from perfect: the 3D-structure based ΔΔG predictors show modest performance even using 3D structures from PDB [26], with correlation of 0.59 or less in independent tests [27]. Thus, using AlphaFold models instead of PDB structures does not make ΔΔG predictions more accurate [15], so availability of AlphaFold models is expected to show an approximately 0.59 correlation with predictions of ΔΔG, which may be too low for many applications.

The deep learning approach demonstrated by AlphaFold may be an inspiring example to develop a deep learning ΔΔG predictor. However, we see the dramatic difference between the situations with 3D structure prediction and ΔΔG prediction that may impede this development. The difference is in the amount of available data. For protein structure prediction AlphaFold used PDB with ∼150,000 files, and each file contained a wealth of information. In contrast to PDB, the number of experimentally measured ΔΔG values are of the order of 10,000 and these are just numbers without accompanying extra data. To make a rough comparison of information in bits, PDB structures occupy 100 Gb, while all the known experimentally ΔΔG values occupy about 10 kb. Neural networks are very sensitive to the amount of information in the training set so the ability of deep learning to tackle the ΔΔG prediction task at present looks hindered mostly by the lack of experimental data.

Overall, we explored the capacity of direct prediction of ΔΔG by all AlphaFold metrics reported in the standard deafault mode: (i) the difference in the pLDDT score before and after mutation in the mutated position, (ii) the difference in the averaged pLDDT score across all positions before and after mutation. We found that the correlation was weak or absent, and, therefore, AlphaFold predictions are unlikely to be useful for ΔΔG predictions. Taken together with our recent result that AlphaFold models are not better for ΔΔG predictions than best templates [15], we see no straightforward way to use AlphaFold advances for solving the task of prediction of ΔΔG upon mutation. The task of ΔΔG prediction should be solved separately and it will face the problem of limited amount of data for training neural networks.

## Supporting information

S1 Table List of studied mutations with ΔΔG values. The list of 1154 mutations in 73 proteins randomly chosen for ΔΔG analysis as described in Materials and methods).
(XLSX)

S2 Table List of studied single mutations in GFP. The list of 796 single mutants of GFP randomly chosen for our analysis as described in Materials and methods).
(XLSX)

S3 Table Correlations for mutation categories. Pearson (PCC) and Spearman (SCC) correlation coefficients for different categories of mutations. P-values were calculated by Kruskal-Wallis H-test.
(XLSX)
